# A novel three-dimensional echocardiographic method for device size selection in patients undergoing ASD trans-catheter closure

**DOI:** 10.1186/s43044-019-0038-7

**Published:** 2019-12-31

**Authors:** Alaa Roushdy, Aya El sayegh, Yasmin Abdelrazek Ali, Hebattalla Attia, Azza El fiky, Maiy El sayed

**Affiliations:** Cardiology Department, Congenital and structural heart diseases unit, Ain Shams University hospitals, Cairo, Egypt

**Keywords:** ASD, Device closure, three-dimensional echocardiography

## Abstract

**Background:**

Proper device size selection is a crucial step for successful ASD device closure. The current gold standard for device size selection is balloon sizing. Balloon sizing can be tedious, time consuming and increase fluoroscopy and procedure times as well as risk of complications. We aimed to establish a simple and accurate method for device size selection using three-dimensional echocardiographic interrogation of the ASD.This is a prospective observational study conducted over a period of 12 months. All patients underwent 2D TTE, three-dimensional echocardiographic assessment of the IAS and transesophageal echocardiogram. Comparison between echocardiographic variables was done using independent sample t test. Linear correlation was established between three dimensional echocardiographic variables and respective variables of device size and 2D TTE and TEE measurements.

**Results:**

The study included 50 patients who underwent successful ASD device closure with properly sized device. There was no significant difference between 3D ASD maximum diameter and all diameters measured by TTE and TEE. There was a strong positive correlation between device size used for closure and both 3D measured ASD area (r = 0.907, P<0.0001) and 3D measured ASD circumference (r = 0.917, P<0.0001). Two regression equations were generated to determine proper device size where Device size = 10.8 + [3.95 x 3D ASD area] and Device size = [3.85 x 3D ASD circumference] -1.02

**Conclusion:**

Three-dimensional echocardiogram can provide a simple and accurate method for device size selection in patients undergoing ASD device closure using either 3D derived ASD area or ASD circumference

## Background

Trans-catheter atrial septal defect (ASD) closure has been widely used as an alternative to surgical closure in specific cases with excellent long-term results. Selection of the appropriate device size is considered the corner stone for successful procedure, as under- or over sizing may cause complications such as device embolization or laceration of adjacent structures [1]. Using balloon sizing to determine the device size for trans-catheter ASD closure has been regarded as the gold standard. However, several studies have been conducted to avoid balloon sizing during the intervention, as it prolongs the procedure and fluoroscopy time and may also cause complications [2].

Assessment of ASD morphology and relationship with near-field structures was enhanced through the introduction of three-dimensional echocardiography [3]. Initially three dimensional (3D) measurements were complex, time taking and difficult to perform. Acquisition and post-processing were time consuming and not applicable during catheterization [4]. The development of matrix ultrasound probes allowed the visualization of a real-time 3D cardiac image within a user defined volume, in a shorter time and more simple steps. The miniaturization of the 3D transducer allowed its incorporation into trans-esophageal (TEE) ultrasound probes. In the catheterization laboratory, 3D-TEE now plays a crucial role in 3D real-time guidance of percutaneous closure [5].

We sought to correlate data obtained from 3D assessment of the ASD, with conventional measurements of ASD as well as device size, device circumference and device area used for ASD closure. Accordingly, we conducted this prospective study to establish a simple and accurate method for device size selection using three-dimensional echocardiographic interrogation of the ASD.

## Methods

This study was approved by our institutional review board, and informed consent was obtained from all enrolled patients.

### Study population

This was a prospective observational study which initially included 64 patients who were referred for elective ASD device closure over a period of 12 months.

Patients with any of the following criteria were excluded from the study: (1) Primum ASD (2) Eisenmenger syndrome (3) associated congenital anomalies requiring surgical intervention (4) Secundum ASD with unsuitable anatomy for closure due to defective or floppy rims diagnosed by transthoracic or transoesophogeal echocardiography (5) Secundum ASD larger than 36 mm in diameter (6) Patients with inter atrial septal aneurysm or fenestrated septum with multiple defects for which 3D tracing of the defect to produce 3D derived area and circumference deemed impossible or inaccurate.

## Methods

All patients enrolled in the study were subjected to the following:

### Two-dimensional transthoracic echocardiogram

Full transthoracic echocardiogram using a Philips iE33 machine (Philips Medical Systems, Andover, MA). Standard 2D echocardiogram was done for all patients enrolled in the study using phased array transducers of different frequencies tailored according to each patient’s age, body built and weight.

The study included 2D, M-mode and color flow Doppler from all standard echocardiographic windows (i.e. sub costal, apical, parasternal and supra sternal) applying the sequential analysis to detect any associated congenital anomalies. Three views were used to visualize the inter-atrial septum, the sub costal biatrial view, the sub costal sagittal or bicaval view and parasternal short axis view. Two dimensional (2D) and color flow mapping were used to evaluate the site and diameter of the ASD in the 3 views [6]. The maximum diameter in any of the 3 views was recorded as the maximum transthoracic echocardiographic (TTE) diameter of the ASD.

Patients deemed suitable for ASD device closure were then subjected to the following:

### Three-dimensional echocardiogram [6]

After completing the 2D echocardiography, all subjects underwent 3D echocardiogram study by an independent operator blinded to the data obtained by both TTE and 2D TEE. In patients, less than 20 kg body weight a 3D TTE data were obtained using the Vivid 9 GE machine 4V probe and for patients more than 20 kg 3D TEE data were obtained using the vivid 9 GE machine TEE 6VT probe.

We used the 3D zoom prepare modality for image acquisition. In 3D TEE, the bicaval view was acquired at the mid-esophageal level with the transducer starting at the 90 to 120 degrees. The depth of pyramidal data sets was adjusted to include only the left and right sides of the atrial septum in this view to allow the entire septum to be acquired in a 3D format without incorporating the surrounding structures. With a 90 degree up–down angulation of the pyramidal data set, the entire left-sided aspect of the septum could be shown in an “en face perspective”.

**Fig. 1:**
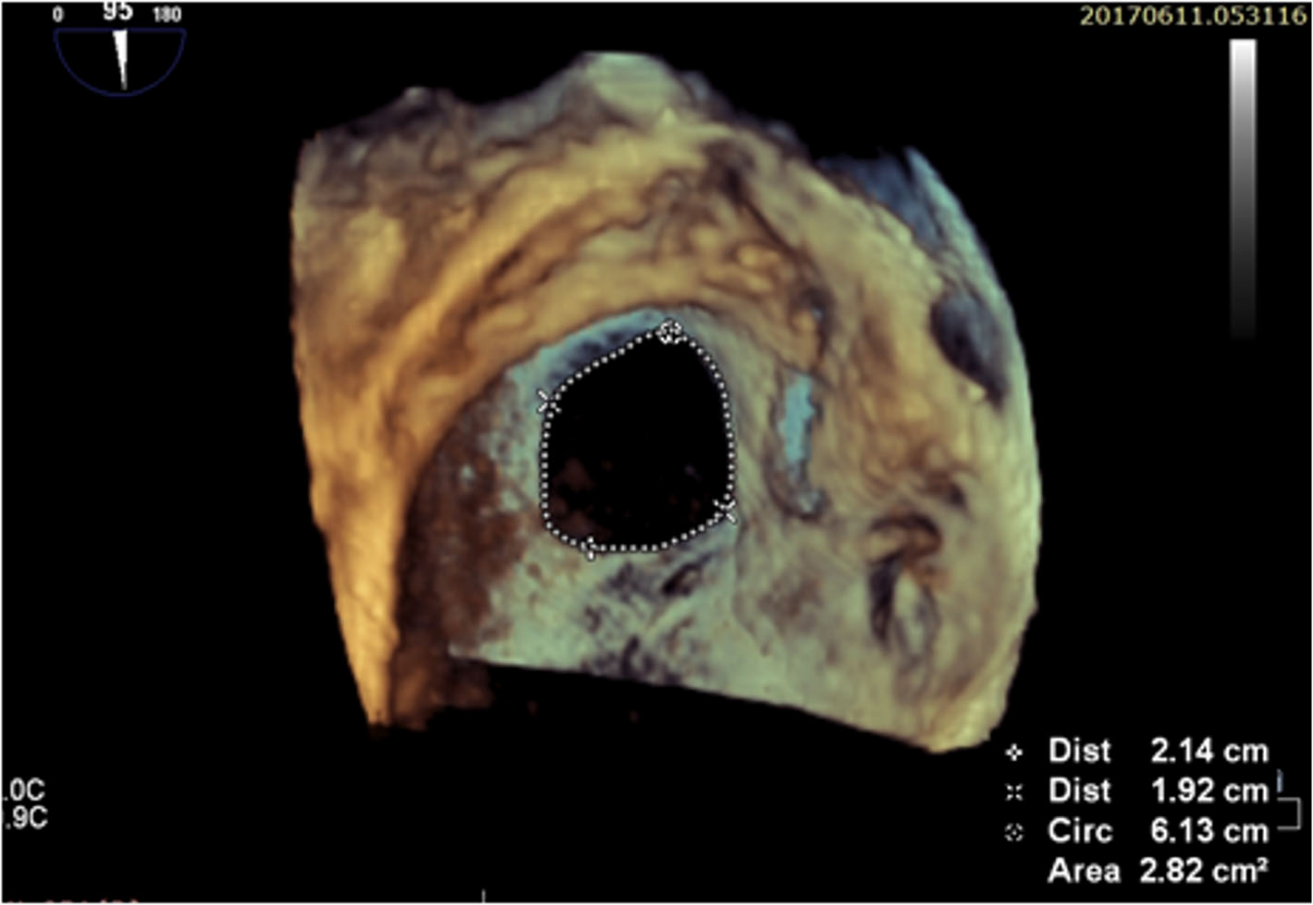
3D TEE image of the patient showing ASD dimensions, area and circumference measurements all visualized from the RA side

Once the left side of the atrial septum has been acquired, a rightward tilting of the volume will show the right side of the atrial septum and the fossa ovalis as a depression on the septum. In some cases, fine cropping using the arbitrary crop plane was necessary to remove the surrounding atrial structures obscuring the septum. A gain setting at medium level was required to avoid the disappearance of the fossa ovalis and creating a false impression of an ASD (Fig. 1).

**Fig. 2:**
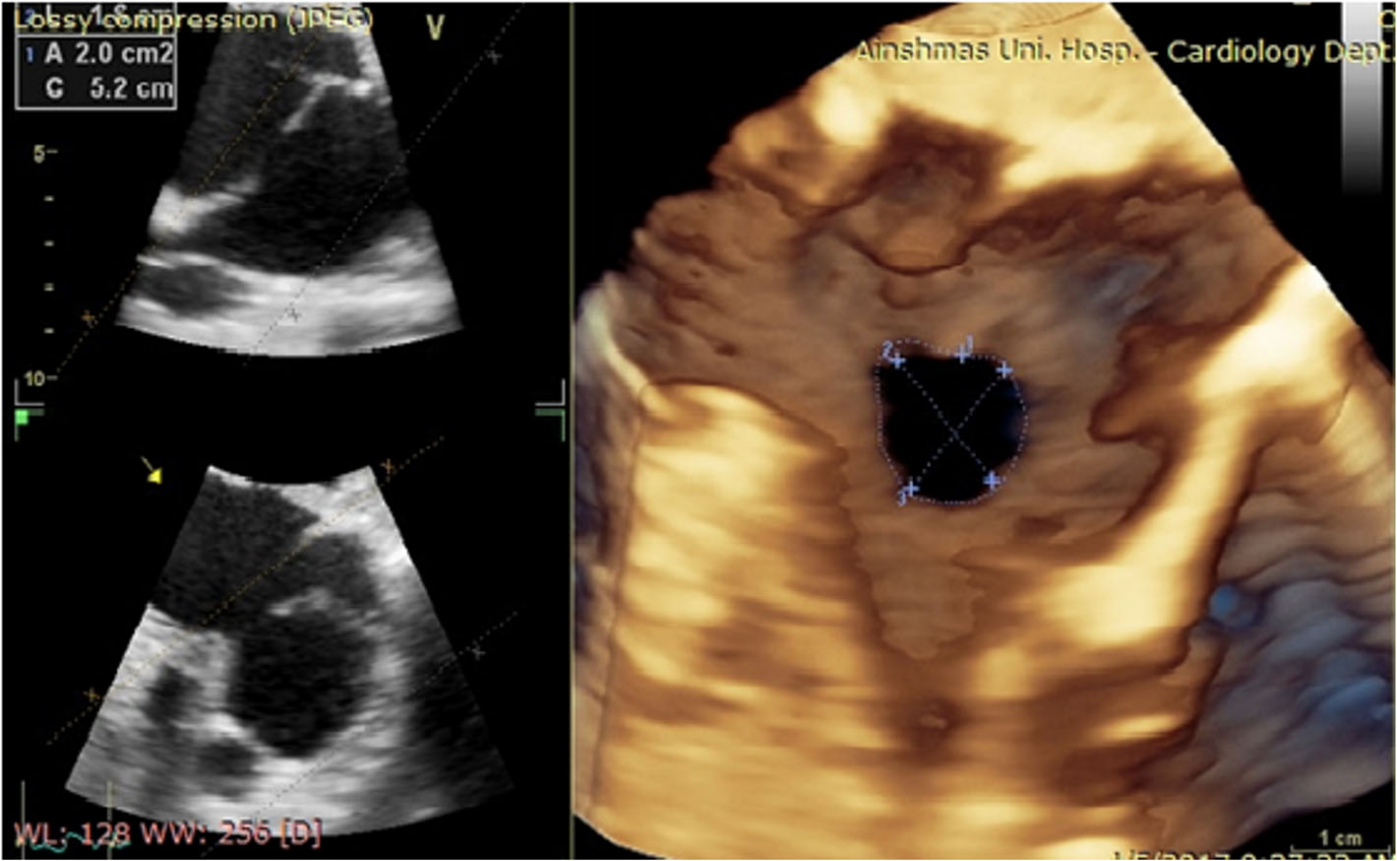
3D TTE of the patient showing central moderate rounded secundum. ASD with dimensions, area and circumference measurements all visualized from RA side

For 3D TTE image acquisition, we used the 3D zoom prepare in the sub-coastal window, bicaval view then the volume was oriented to view the IAS from the RA with the SVC located at the 11-o’clock position (Fig. 2).

In terms of data analysis, after adjusting the sector aiming for the septum to be in the “en face view”, excluding possibility of oblique view, the defect area and circumference were measured by ball tracking the defect, the defect shape, position and 2 dimensions were documented in the 3D image.

### Two dimensional transoesophogeal echocardiogram

2D TEE was done on the day of the procedure inside the cath. Lab. under general anesthesia.

Five key views were used to assess the IAS and surrounding structures these views include the upper esophageal short-axis view, mid esophageal aortic valve short-axis view, four-chamber view, bicaval view, and long-axis view.

Thorough evaluation of the rims surrounding the defect was done. A deficient rim was defined as less than 5 mm in at least three sequential related multi plane views in 15 degrees increments [6]. The presence of normal pulmonary venous drainage was confirmed and the presence of a fenestrated defect or other anatomical variants was also recorded.

The maximum ASD diameter defined as the largest diameter measured between 2 stable rims in any of the recorded views was used for further analysis.

### Trans-catheter ASD closure

All procedures were done under general anesthesia. Right femoral vein access was established using Sildenger’s technique. After crossing the defect, multipurpose catheter was advanced into the upper left pulmonary vein and 0.35/260 stiff guidewire positioned in that vein. Balloon sizing of the defect was done in defects which were seen to have floppy rims by 2D TEE. The balloon stretched diameter was measured by both cine recording and TEE. The delivery sheath was then advanced over the wire. The device was then loaded and advanced to the tip of the sheath and deployed using standard technique. In some patients with difficult alignment of the device along the IAS; right upper pulmonary vein approach or balloon assisted technique was used.

### Device measurements

Device size selection was 1-4 mm larger than the maximum diameter measured by TEE depending on the stability of the ASD rims and whether or not a balloon sizing was used. Due to the circular nature of the device waist, device area and device circumference were calculated as: device area = μr^2^ and device circumference = 2 μr respectively where the device radius (r) was calculated as device size/2.

### Statistical analysis

All data were gathered, tabulated, and statistically analyzed on a PC using a commercially available statistical software package MedCalc version 11.6.1.0 (MedCalc Software, Mariakerke, Belgium). Qualitative variables were expressed as frequencies and their related percentage. Quantitative variables were expressed as mean ± SD. Independent sample T test was used to compare different quantitative variables. Pearson linear correlation was used to determine the correlation between three dimensional measurements and measurements obtained by two dimensional echocardiogram as well as device size, device area and device circumference. Regression analysis was used to generate two equations to predict optimal device size for ASD closure based on 3D derived ASD area and 3D derived ASD circumference. *P*-value was considered significant if <0.05, and *P*<0.01 was considered highly significant.

## Results

The study included 50 patients who underwent successful ASD device closure over a period of 12 months. Procedural success was defined as the presence of all 3 following criteria: successful device delivery without periprocedural complications, well-positioned device, with no device migration; and hospital discharge on postprocedure day 2. We included only cases with the proper device size selected. Proper device size selection was defined as absence of significant residual shunt around free edge of device (to exclude under sizing), no encroachment to surrounding structures with proper re-shaping in correct profile (to exclude oversizing)

Clinical and demographic characteristics of the study group are listed in Table 1.

**Table 1:** Clinical and demographic characteristics of the patients

Characteristics	Findings
Gender (M/F)	18/32
Mean age in years	19.3 +/- 14.09
Mean weight in Kg	48.92 +/- 27.6
Mean height in cm	139.2 +/- 30.52
Mean BSA in m^2^	1.35 +/- 0.526
Associated cardiac anomalies	
None	(n=47)(96%)
AR	(n=1)(2%)
MVP/MR	(n=1)(2%)
PS	(n=1) (2%)
Mode of presentation	
Accidentally discovered	(n=23) (46%)
Shortness of breath	(n=18) (36%)
Palpitation	(n=5) (10%)
Atypical chest pain	(n=4) (8%)

BSA= Body surface area, AR= Aortic regurgitation, MVP= Mitral valve prolapse, MR= Mitral regurgitation, PS= Pulmonary stenosis

**Fig. 3:**
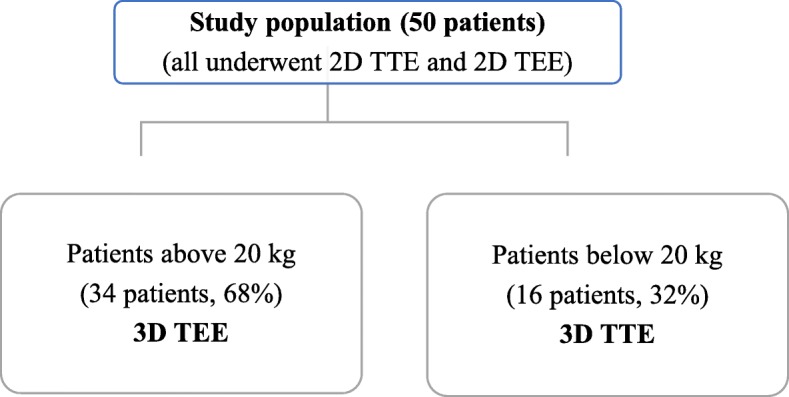
Study population. (2D TTE: 2dimensional transthoracic echocardiography, 2D TEE: 2dimensional transesophageal echocardiography, 3D TTE: 3dimensional transthoracic echocardiography, 3D TEE: 3dimensional transesophageal echocardiography)

All patients included in the study underwent 2D TTE and TEE, thirty-four patients (68%) were evaluated by 3D TEE and sixteen patients (32%) were evaluated by 3D TTE (Fig. 3). The mean ASD diameter measured by 2D TTE was 15.95 +/- 5.82 mm in sub costal sagittal view, 15.85 +/- 5.99 mm in sub costal biatrial view and 14.58 +/- 7.01 mm in parasternal short axis view, while the mean value for maximum ASD diameter in 2D TEE was 16.74 +/- 6.33 mm. The mean maximum and minimum ASD diameters measured by 3D echocardiogram were 17.7 +/- 6 mm and 14.47 +/- 6 mm respectively.

Among the study population, twenty patients (40%) had adequate all rims, thirty patients (60%) had deficient aortic rim. Twenty-three patients (46%) had floppy rims for which balloon sizing was needed. The mean balloon stretched diameter for these patients was 19.6 +/- 5.36 mm. There was no significant difference between 3D echo maximum ASD diameter measured and all the 2D echo diameters as well as the balloon stretched diameters in selected cases with the exception of the parasternal short axis diameter measured by 2D TTE (P= 0.02) (Table 2). There was no significant difference between the 2D TTE ASD diameter measured in the parasternal short axis view and the minimum diameter measured by 3D echocardiogram (P=0.93).

**Table 2:** univariate analysis of 3D echo maximum ASD diameter versus 2D echo ASD diameters using independent sample T test

General	GROUP TYPE						Paired T test	
	3D echo maximum diameter			2D echo diameters			t	P
Sub costal biatrial diameter	17.70	+-	6.03	15.85	+-	5.99	-1.539	0.1270
Sub costal sagittal diameter	17.70	+-	6.03	15.96	+-	5.83	-1.465	0.1462
Parasternal short axis diameter	17.70	+-	6.03	14.58	+-	7.02	-2.355	0.0206
2D TTE maximum diameter	17.70	+-	6.03	17.26	+-	6.52	-0.348	0.7289
2D TEE maximum diameter	17.70	+-	6.03	16.74	+-	6.33	-0.779	0.4376
Balloon stretched diameter	17.70	+-	6.03	19.60	+-	5.37	1.290	0.2013

There was a strong highly significant correlation between different ASD diameters measured by 2D echocardiographic views and that measured by 3D echocardiogram. The strongest correlation was between 3D echocardiogram ASD maximum diameter and 2D TEE ASD maximum diameter (r=0.902, P<0.0001) (Table 3). There was also strong highly significant correlation between 3D ASD diameters and balloon stretched diameters in patients who underwent balloon sizing (r= 0.894, 0.885, P<0.0001)

**Table 3:** correlation between maximum and minimum ASD diameter measured by 3D echocardiogram and diameters measured by 2D TTE and 2D TEE

		2D TTE ASD PSSX	2D TTE ASD biatrial	2D TTE ASD sagittal	2D max diameter	TEE max diameter
3D d1	r	0.829	0.790	0.723	0.780	0.895
	P	<0.0001	<0.0001	<0.0001	<0.0001	<0.0001
3D d2	r	0.871	0.826	0.730	0.792	0.902
	P	<0.0001	<0.0001	<0.0001	<0.0001	<0.0001

r= Pearson correlation coefficient

In terms of the ASD shape evaluated by 3D imaging, twenty –seven patients (54%) had rounded defects, seventeen patients (34%) had oval defects and six patients (12%) had irregular defects.

A wide range of ASD occluder device sizes were used to close the ASD among the study group ranging from 11 to 40 mm device with a mean device size of 19.74 +/- 6.6. There was significant difference between 3D measured defect area and that of the device waist area (P=0.005). There was also significant difference between the 3D measured defect circumference and the device circumference (P<0.0001) (Table 4). The waist area oversized the actual 3D measured defect area by 50% despite the fact that device size in the cath. Lab was chosen as 1-4 mm more than the largest diameter measured by 2D TTE in patients with steady rims or equal to the balloon stretched diameter in patients where balloon sizing was needed. This was equal to 1.16+/- 0.09 the maximum ASD diameter measured.

**Table 4:** univariate analysis of 3D echo guided defect area and circumfrence versus device parameters using independent sample T test

General	GROUP TYPE						Paired T test	
	3D echo measurements			Device measurements			t	P
3D defect area versus device waist area in cm^2^	2.25	+-	1.53	3.38	+-	2.31	2.854	0.0053
3D defect circumference versus device waist circumference in cm	5.36	+-	1.66	2.07	+-	3.45	11.291	< 0.0001
3D defect area versus left atrial disc area in cm^2^	2.25	+-	1.53	9.16	+-	4.02	2.181	0.0316
3D defect circumference versus left atrial disc circumference in cm	5.36	+-	1.66	2.25	+-	6.90	12.815	< 0.0001

**Fig. 4:**
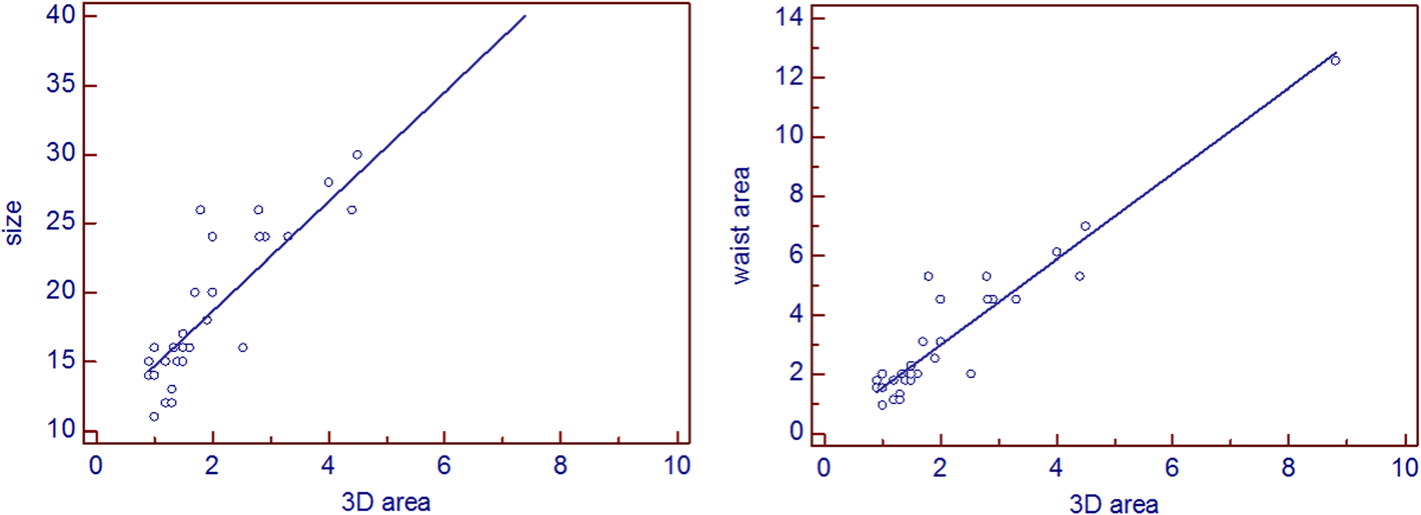
Linear correlation showing a strong significant correlation between 3D derived defect area and device size used for ASD transcatheter closure (left panel) and device waist area (right panel)

**Fig. 5:**
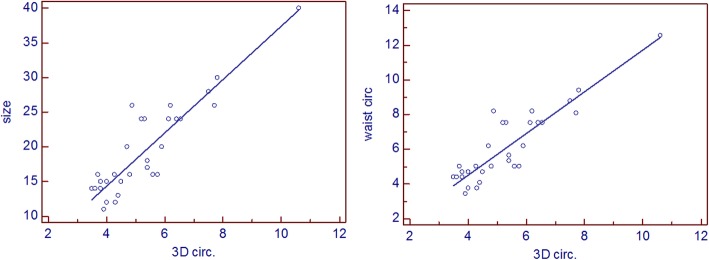
Linear correlation showing a strong significant correlation between 3D derived defect circumference and device size used for ASD transcatheter closure (left panel) and device waist circumference (right panel)

Nevertheless there was strong highly significant correlation between 3D derived defect area and both device size implanted for the patients (r = 0.907, P<0.0001) and device waist area (r = 0.945, P<0.0001) (Fig. 4). There was also strong highly significant correlation between 3D derived defect circumference and both device size implanted for the patients (r = 0.917, P<0.0001) and device waist circumference (r = 0.915, P<0.0001) (Fig. 5). Based on these correlations two regression equations were derived to predict the suitable device size based on either 3D derived defect area and 3D derived defect circumference where:

1 $$ \mathbf{Devicesize}=\mathbf{10.8}+\left[\mathbf{3.95}\mathbf{x}\mathbf{3}\mathbf{DASDarea}\right] $$

2 $$ \mathbf{Devicesize}=\left[\mathbf{3.85}\mathbf{x}\mathbf{3}\mathbf{DASDcircumference}\right]-\mathbf{1.02} $$

The mean predicted device size determined by regression analysis was 19.71 and 19.6 using equation 1 and equation 2 respectively. There was no significant difference between these measurements and actual mean device size implanted which was 19.745 (P = 0.94).

## Discussion

The unique ability of 3D echocardiogram to visualize the interatrial septum and any related ASD in “en face view” ensure proper measuring of any ASD far beyond the capability of any existing imaging modality. We tried to make use of this feature to produce a simple measurement through which the optimal device size can be predicted based on 3D measured ASD area or circumference.

Sixty-four percent of our patients were females, with mean age at closure of nineteen years. This finding coincides with the fact that secundum ASD has a female predominance of approximately 2:1, and is frequently diagnosed in adulthood [7]. Sixty percent of our study population had deficient retro aortic rim. Similarly, Petit and colleagues found a prevalence of deficient aortic rim of 59% [8]. O’ Byrne and colleagues concluded that deficient retro-aortic rim was highly prevalent [9].

In the current study the TEE maximum ASD diameter was higher than the maximum ASD diameter measured by TTE, however there was strong positive correlation (r=0.797, P value<0.0001) between both measurements, this was similar to the results found by Se’bastien Hascoet and colleagues [10].

The maximum ASD diameter measured by 3D imaging and TEE maximum diameter values showed strong positive correlation (r= 0.895 and P value < 0.0001). This was similar to the results found by Jeong-Sook Seo who enrolled 107 patients with secundum ASD for device closure and found that the maximal and minimal diameters measured using 3D-TEE showed excellent correlations with those using 2D-TEE (r = 0.954, r = 0.906, P < .001 respectively) [11].

Device size selection was 1-4 mm larger than the maximum diameter measured by TEE depending on the stability of the ASD rims and whether or not a balloon sizing was used, this represent a 1.16+/- 0.9 the maximum diameter of the defect measured by 2D TEE which was in accordance to general consensus of choosing ASD device equals 1.2 the maximum diameter of the defect measured by 2D TEE used by Bartakian et al. [12].

The significant difference between the waist area and circumference of the selected ASD device and that measured by 3D echocardiogram may be explained by the fact that the current consensus in choosing a suitable device for trans-catheter ASD closure uses 2D TEE measurements to examine a 3D structure. Also a considerable number of ASDs are actually elliptical more than circular, a fact which could not be assessed except by 3D echocardiogram.

Despite this significant difference we found very strong positive correlation between 3D defect area and device waist area (r=0.954 and P value<0.0001), and between 3D defect circumference and device waist circumference (r=0.915 and P value < 0.0001). Based on these correlations linear regression analysis was used to generate an equation through which device size could be estimated using either 3D area or 3D circumference. To the best of our knowledge, this is the first study to generate simple equations to predict device size using direct measurement of 3D defect area and 3D defect circumference, no previous studies in the literature demonstrated this relation. Previous studies done using 3D derived dimensions to predict the optimum device size were very limited and generated more complex equations.

Jeong-Sook Seo and colleagues prospectively enrolled 107 consecutive patients who underwent trans-catheter closure of ASD, They assessed the relationship between the balloon stretched diameter and the diameters measured using 2D and 3D trans-esophageal echocardiography and they suggested that:

$$ \mathbf{Device}\ \mathbf{size}=\left(\mathbf{0.964}\ \mathbf{x}\ \mathbf{3}\mathbf{D}\ \mathbf{\max}\right)-\left(\mathbf{2.622}\ \mathbf{x}\ \mathbf{circular}\ \mathbf{index}\right)+\mathbf{7.084} $$

Where: 3D max and the circular index are the maximal diameter on 3D imaging and the ratio of the maximal diameter to the minimal diameter on the 3D-TEE image [11]. Unlike our study, the authors of this study generated there equation based on using balloon stretched diameter for all patients enrolled in the study, which is not an everyday practice and generated more complex equation than the ones presented in the current study.

Se’bastien Hascoet et al. [10] enrolled 30 children who underwent percutaneous closure of a single ASD and using multivariate linear regression analysis, two formulas were built to predict balloon stretched diameter (BS)

The first model was:

$$ \mathbf{BS}=\left(\mathbf{1.07}\times \mathbf{3}\mathbf{D}-\mathbf{TEEmax}\right)-\left(\mathbf{3.1}\times \mathbf{ASD}\ \mathbf{shape}\right)+\mathbf{3}. $$

The ASD shape was 0 for round and 1 for oval ASDs.

A second model was:

$$ \mathbf{BS}=\mathbf{4.5}\times \mathbf{ASD}\ \mathbf{area}+\mathbf{11.5}. $$

Again these equations were based on predicting the balloon stretched diameter and not the exact device size and the authors used device shape in one model which may be subjective in some cases.

### Study limitations and recommendations

Limitations of 3D echocardiography should be taken in consideration. Including need for proper gain acquisition to avoid under or overestimation of measurements of the defects. 3D TEE helped to overcome the limitation of unavailable acoustic window in comparison to 3D TTE, yet it can be performed only in patients more than 20 kg.

We recommend validation of the equations generated in this study using a validation group to test the clinical applicability of these equations to predict proper device selection in patients undergoing trans-catheter ASD closure. Such a validation can represent an important step to allow for future development of automated 3D software that can predict device size in cases of ASD device closure similar to the currently available software for mitral valve quantification or those under development for TAVI using 3D echocardiogram.

A safety and efficacy study including 2 tiers of patients is also recommended. In the first tier, the device size should be selected using the equation developed in the current study to generate pre-determined device size, in the second-tier device size selection will be based on the classic measurements including 2D TTE ,2D TEE and balloon sizing to compare both tiers as regard procedure time, fluoroscopy time, safety and efficacy

## Conclusions

A 3D echocardiographic measurement of different ASD parameters is feasible and accurate using either 3D TTE in smaller children or 3D TEE for older patients. Strong highly significant correlations exist between 3D derived ASD area and circumference and device size used for ASD closure. 3D derived equations based on these correlations to predict proper device size needed for ASD closure based on 3D derived ASD area or circumference represent a simple and novel technique that can increase safety and efficacy of ASD device closure.

## Data Availability

The datasets used and/or analyzed during the current study are available from the corresponding author on reasonable request.

## Abbreviations

2D: Two dimensional
3D: Three dimensional
ASD: Atrial septal defect
BS: Balloon stretched
TEE: Transoesophogeal echocardiography
TTE: Transthoracic echocardiography
